# Spectrum and antibiogram of bacteria isolated from patients presenting with infected wounds in a Tertiary Hospital, northern Tanzania

**DOI:** 10.1186/s13104-017-3092-9

**Published:** 2017-12-20

**Authors:** Nancy A. Kassam, Damian J. Damian, Debora Kajeguka, Balthazar Nyombi, Gibson S. Kibiki

**Affiliations:** 1 Kilimanjaro Christian Medical University College (KCMUCo), P.O. Box 2240, Moshi, Tanzania; 2 Kilimanjaro Christian Medical Centre (KCMC), P.O. Box 3010, Moshi, Tanzania; 3 Kilimanjaro Clinical Research Institute (KCRI), Moshi, Tanzania

**Keywords:** Wound infection, Drug resistance pattern, ESBL, Antibiogram, Surgical site infection, Diabetic wounds, Trauma wounds

## Abstract

**Objective:**

This study aimed to determine the spectrum and antibiogram of the isolated bacteria from patients presenting with infected wounds at Kilimanjaro Christian Medical Centre in northern Tanzania.

**Results:**

Bacterial growth was observed in the vast majority of wound swabs (91.4%). Most of the bacteria isolated (62.3%) were Gram-negative rods. *Staphylococcus aureus* was the most common isolated organism (16%) followed by other Coliforms and *Enterococcus* spp. (12.5% each). *Enterococcus* spp. (36.4%) was the most common isolated bacteria in diabetic wounds whereas *S. aureus* was the most common isolated bacteria from the wounds caused by trauma (40.0%) and surgical site infection (20.6%). Resistance was high to most common antibiotics used in the hospital.

**Electronic supplementary material:**

The online version of this article (10.1186/s13104-017-3092-9) contains supplementary material, which is available to authorized users.

## Introduction

Bacterial infections of wounds are among the leading causes of morbidity and mortality throughout the world and are regarded as one of the most common nosocomial infections. Wound infections have been reported to vary between 3 and 11% in developed countries and estimated to be as high as 40% in developing countries [1–3]. Wound infections increase with the degree of wound contamination, and it is estimated that 50% of wounds contaminated by bacteria become clinically infected [4].

Drug resistance impinges on the quality of patient care through its associated mortality, morbidity and significant economic consequences [5]. In hospital practice, 30–50% of antibiotics are prescribed for surgical prophylaxis and 30–90% of these prophylaxes are inappropriate [6]. Inappropriate use of antibiotics increases selection pressure favouring the emergence of pathogenic drug-resistant bacteria which makes the choice of empirical antimicrobial agents more complicated [7, 8].

Extended spectrum beta-lactamase (ESBL) producing organisms are another type of common bacteria resistant to antibiotics. ESBL producing Gram-negative rods (GNRs) have spread all over the world [8, 9]. The prevalence of ESBL producing GNR varies across the world from 50 to 80% [8, 10, 11]. About 33% of infections by ESBL producers are deadly. In Tanzania, the death rate due to ESBL producing GNR is as high as 13.9% [12].

Comparing to Gram-negative, Gram-positives bacteria have been reported to be less prevalent causing wound infections [8, 11, 13–15]. *Staphylococcus aureus* (*S. aureus*) has been reported to be the most common isolated bacteria from different wound types. *Pseudomonas aeruginosa* are commonly isolated in infected wounds following surgeries and burns whereas *Enterococcus* species and *Enterobacteriaceae* are commonly isolated from wounds in immune-compromised patients and abdominal surgeries [4, 8, 16–18].

The majority of the isolates from infected wounds are known to be resistant to ampicillin and amoxicillin. Large numbers of *S. aureus* are methicillin-resistant *S. aureus* (MRSA) and most bacteria isolated are sensitive to quinolones, aminoglycosides and monobactam [10, 11, 19–21].

Infection in a wound delays healing, prolongs hospital stay, increases trauma, poses risk for disarticulation and amputation, increases need for medical care and increases treatment costs [22]. This makes infection of wounds a matter of concern and makes it necessary to study the causative agents of these infections and their antibiogram.

## Main text

### Characteristics of participants and enrolment procedures

Patients with Surgical Sites Infections (SSI), infected diabetic wounds, infected wounds due to trauma, and patients with other infected wounds admitted in surgical ward at Kilimanjaro Christian Medical Centre (KCMC) from July 2013 to June 2014 were included in this study. Prior to enrolment in the study, patients were examined by a physician for a suspected or actual wound sepsis using the following criteria; ‘cellulitis’, ‘maladour’, ‘pain’, ‘delayed healing’, ‘deterioration in the wound’ or ‘wound breakdown’ and ‘increase in exudate volume’. Patients presenting with at least three of these clinical signs were enrolled in the study. Chronic wound was differentiated from acute wound if it failed to heal within 4 weeks and showed no sign of improvement within 8 weeks.

### Data collection

#### Pus swabs collection and culture

Wound swabs were collected from patients with infected diabetic wounds, surgical sites, trauma and other wounds by the research nurse. To avoid contaminating the swab with skin flora, pus or necrotic tissue, the wound was thoroughly cleansed with 60–120 mL sterile normal saline prior to taking the sample. Sterile gauze was used to remove excess saline from the wound surface and the pus swabs were collected using sterile swab by swabbing at the middle of the wound. When there were two or more wounds in the same location, separate swabs were used for each wound. A swab moistened with sterile normal saline was rolled deep in the wounds and inserted immediately into a tube containing Stuart’s transport media for preservation of microbes and then transported to the laboratory [8, 16]. Pus swabs were streaked on Blood Agar (BA) and MacConkey Agar (MCA) plates and incubated aerobically for 18–24 h at 37 °C. They were then observed for bacterial growth. Plates with no growth and with growth were re-incubated for another 18–48 h for isolation of bacteria that require extended incubation (slow growers) [8, 16, 17].

#### Identification of bacterial pathogens

Standard techniques were used for identification of pathogenic bacteria isolated in pure cultures. Characteristic morphological appearances of colonies on media, Gram stains and standard biochemical tests including catalase, coagulase, oxidase, Voges Proskauer, hydrogen sulphide production, urease, methyl red, indole, citrate, CAMP test and sugar utilisation were used to characterise bacteria and identify them [17].

#### Antibiotics susceptibility testing

Drug susceptibility tests were performed using the Kirby–Bauer disk diffusion method according to Clinical and Laboratory Standards Institute (CLSI) guidelines. A sterile swab was dipped into the suspension of the isolate in normal saline, squeezed free from excess fluid against the side of tube and spread over the Mueller–Hinton agar plate. The density of suspension to be inoculated was determined by comparison with the optical density of McFarland 0.5 Barium sulfate solution. Sensitivity discs of appropriate antibiotics were placed onto the media and incubated at 37 °C for 16–18 h except for *coagulase*-*negative staphylococci* which was incubated for 24 h and *methicillin*-*resistant staphylococci* at 35 °C [23]. Zones of inhibition were read and, incubation and resistance rates to respective antibiotics were determined.

#### ESBL production screening

ESBLs production was tested by the disc diffusion method on Mueller–Hinton Agar according to the CLSI guidelines and confirmed by the double disc approximation method [23].

### Statistical analysis

Clinical, demographic and laboratory data were entered and linked for each patient using Statistical Package for Social Science software version 20 (IBM Corp, Chicago IL). Thereafter, data were cleaned and analysed using Stata software (Version 13, StataCorp, College Station, Texas). Numeric variables were summarised using measures of central tendency with their respective measures of dispersion while frequency and percentages were used to summarise categorical data.

## Results

### General characteristics of the study participants

In total, 93 patients diagnosed with infected wounds were enrolled in this study. Male patients numbered 63 (67.7%). The median (range) age at recruitment was 45 (1–80) years. Most of participants, i.e. 65 (71.4%) had acute wounds. The majority of patients, i.e. 82 (90.1%) indicated to have used antibiotics either as a prophylaxis or treatment previously. Additional file 1: Table S1 shows these results.

### Bacteria isolated

**Fig. 1:**
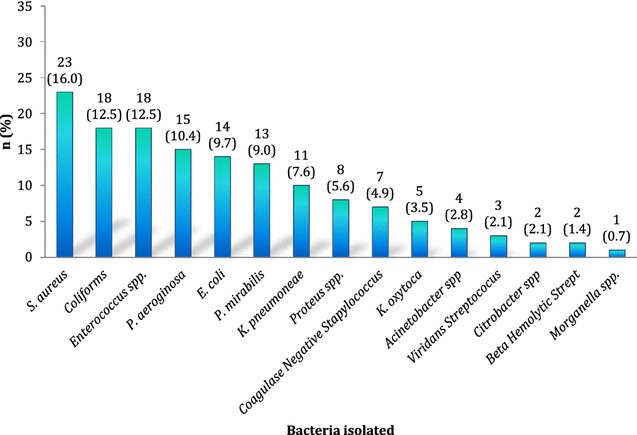
Species of bacteria isolated

A total of 93 wound swabs from 93 patients were cultured and 146 bacteria were isolated. Of them 91.4% had bacterial growth. Gram stains of pure cultures showed 91 (62.3%) of the isolates were gram-negative rods. A total of 144 pathogenic bacteria were isolated from 83 cultures. *Staphylococcus aureus* was the most common isolate (16.0%) followed by other Coliforms and *Enterococcus* spp. (12.5% each). Figure 1 shows these results.

More than one-third of the wound infections were caused by single isolate, i.e. 38 (44.7%). According to the type of wounds, *Staphylococcus aureus* was the most isolated bacteria in acute wounds (29.1%) followed by *Pseudomonas aeruginosa* (18.2%) and other *Coliforms* (23%). Whereas in chronic wounds, *Proteus mirabilis* (26.9%) followed by *Enterococcus* species and *Escherichia coli* (23.1%) were the most common isolated bacteria. Additional file 2: Table S2 and Additional file 3: Figure S1 depict these results.

### Antibiogram of the isolated bacteria

*Staphylococcus aureus* showed high resistance to amoxicillin (61.9%). Most Gram-negative rods isolated were very resistant to amoxicillin–clavulanate and cotrimoxazole (66.7–100%) respectively. Tables 1 and 2 show these findings.

**Table 1:** Drug resistance patterns of Gram positive isolates

Antibiotics	Gram positive isolate (%)		
	*S. aureus (n* = *23)*	CNS *(n* = *7)*	*Enterococcus* spp. *(n* = *18)*
Amoxicillin	61.9^a^	33.3^a^	35.7^a^
Amoxicillin–clavulanate	NT	57.1	47.1
Ceftriaxone	21.7	33.3^a^	NT
Ciprofloxacin	4.3	0.0^a^	31.3^a^
Gentamycin	17.4	0.0	NT
Clindamycin	14.3^a^	0.0	NT
Erythromycin	45.0^a^	42.9	42.9^a^

NT, not tested; CNS, Coagulase negative *Staphylococcus*

^a^Not all bacteria were tested against a particular drug

**Table 2:** Antibiotic resistance patterns for Gram negative rods isolated (n = 91)

Antibiotics	Gram negative rods isolated (%)						
	K. PN	K. OX	P. MR	P. SP	P. AE	E. CL	O. COL
Amikacin	0.00	0.00	0.00	0.00	0.00	0.00^a^	4.0
Amoxicillin–clavulanate	100.0	100.0	80.0	66.7^a^	100.0	92.9	96.6
Ceftazidime	70.0	20.0	12.5^a^	33.3^a^	25.0	18.2^a^	72.0
Ceftriaxone	70.0	20.0	37.5^a^	22.2	21.4	38.5^a^	64.0
Ciprofloxacin	30.0	20.0	0.00	0.00^a^	0.00	35.7	36.0
Cotrimoxazole	100.0	80.0	66.7^a^	77.8^a^	92.9	84.6^a^	84.0
Gentamycin	50.0	60.0	40.0	22.2	28.5	50.0	52.0
Cefotaxime	66.7^a^	0.00^a^	50.0^a^	55.5^a^	58.33	45.5^a^	70.8^a^

Gram-negative rods isolated: K. PN, *Klebsiella pneumoniae*; K. OX, *Klebsiella oxytoca*; P. MR, *Proteus mirabilis*; P. SP, *Proteus species*; P. AE, *Pseudomonas aeruginosa*; E. CL, *Escherichia coli*; O. COL, Other *Coliforms*; *Acinetobacter* spp.; *Citrobacter* spp.; *Morganella* spp.

^a^Not all bacteria were tested against a particular drug

#### ESBL producing Gram-negative rods

The 44 Gram-negative rods isolated, including *Klebsiella pneumoniae*, *Escherichia coli* and *Proteus* species, were phenotypically tested for ESBL production. Half (50%) of these isolates were ESBL producers. All ESBL-producing Gram-negative rods showed 100% resistance rates to ceftriaxone, cefotaxime and cotrimoxazole. These bacteria showed resistance rates of 60–100% to amoxClav, ceftazidime and gentamycin. All ESBL producing GNR showed no resistance to amikacin.

## Discussion

In this study, Gram-negative rods were the predominant and leading cause of wound infections. These findings are in line with those of previous studies in Asia and other African settings [8, 11, 13, 14]. This might be due to high resistances to antibiotics showed by Gram-negative bacteria compared to Gram-positive isolates, and therefore their persistence in infected wounds. Furthermore, chronic wounds were infected by multiple Gram-negative rods. The multiple bacterial infections in this case might be due to impaired immune responses associated with diabetes. These results are in accord with recent studies in Tanzania where polymicrobial wound infections were reported to be common in diabetic wounds [8, 24].

In general, *Staphylococcus aureus* was the most common bacteria isolated in this study. This is a finding consistent with most studies done across the world [10, 17, 21]. *S. aureus* are normal flora of the skin and anterior nares, therefore they can easily contaminate wounds and cause infections. Moreover, *S. aureus* are known to have a vast number of virulence factors that increase their ability to cause infections when compared to other bacteria. Our findings are contrary to the study conducted in a similar setting where *Pseudomonas aeruginosa* was the common isolate in SSI. These variations could be attributed to several factors including the nature of the surgical site itself, the wound site, the type of prophylactic antibiotics used for infections prevention, the level of nursing care given and the measures taken to prevent nosocomial infections [8, 10].

*Enterococcus* was the most common bacteria isolated in diabetic wounds, perhaps due to their opportunistic pathogen behaviour since lowered immune responses are associated with diabetes. Other common isolates from IDFUs were *Proteus* and *Klebsiella*, which are known to be common isolates in chronic wounds. These bacteria had high rates of ESBL production and showed high multiple drug resistance (MDR) rates. Studies elsewhere have reported similar findings [13–15, 20, 24].

In this study, high drug resistance was observed for amoxicillin–clavulanate and cotrimoxazole. These antibiotics are relatively cheap and readily available. These, together with policies that do not restrict antibiotics accessibility to patients, might have caused the irrational overuse of these drugs which might have led to bacterial resistance. Cephalosporins were ineffective against most Gram-negative rods. This might be due to mutational emergence and the spread of ESBL-producing Gram-negative rods and the extensive use of these antibiotics in both treatment and prophylaxis. In this setting, good responses were seen for ciprofloxacin and amikacin. In our setting, the use of these antibiotics is highly restricted due to their adverse side effects. Ciprofloxacin has been recommended only in certain bacterial infections. Furthermore, amikacin is very expensive in our community and the majority of patients could not afford to use it. The different levels of resistance to cefotaxime and ceftriaxone are surprising, although this may be influenced by the fact that not all isolates were tested against all antibiotics. Other studies showed findings in accordance with these [8, 10, 19].

## Limitation

Despite being a commonly-used, non-invasive and cost-effective method, swabs might provide a poor specimen as compared to needle aspiration or tissue biopsy if not collected appropriately. Improper specimen collection affects the results obtained, often by reflecting normal skin flora and colonizing organisms, making it difficult to decide which organisms are the true pathogens. However, our results are not likely to be affected by this since the wound was cleansed thoroughly prior to swab collection. Moreover, the small sample size did not allow us to conduct advanced statistical analyses which could have potentially strengthened this study.

## Additional files

**Additional file 1: Table S1.** Background characteristics of the study population.

**Additional file 2: Table S2.** Bacteria isolated in different types of wound infection.

**Additional file 3: Figure S1.** Bacteria isolated from acute and chronic wounds.

## Abbreviations

BA: Blood Agar
CLSI: Clinical and Laboratory Standards Institute
ESBL: Extended spectrum beta-lactamase
GNRs: Gram-negative rods
IDFU: Infected Diabetic Foot Ulcer
KCMC: Kilimanjaro Christian Medical Centre
KCMUCo: Kilimanjaro Christian Medical University College
KCRI: Kilimanjaro Clinical Research Institute
MCA: MacConkey Agar
MDR: multiple drug resistance
MRSA: methicillin resistant *Staphylococcus aureus*
SSI: surgical site infection
