# Supplementary material for: Spectrum and antibiogram of bacteria isolated from patients presenting with infected wounds in a Tertiary Hospital, northern Tanzania
Source: BMC Res Notes. 2017 Dec 20;10:757. doi: 10.1186/s13104-017-3092-9 (PMC5738783; doi:10.1186/s13104-017-3092-9)
Supplement: Supplementary file 1 — Additional file 1: Table S1. Background characteristics of the study population. [file 13104_2017_3092_MOESM1_ESM.docx]

# Table S1: Background characteristics of the study population (N=93).

| **Characteristics** | **n (%)** |
| --- | --- |
| ***Sex:*** |  |
| Male | 63 (67.7) |
| Female | 30 (32.3) |
| ***Age (years):*** |  |
| 0 – 5 | 3 (3.2) |
| 6 – 12 | 1 (1.1) |
| 13 – 20 | 11(11.8) |
| 21 – 40 | 27 (29.0) |
| 41 – 60 | 26 (28.0) |
| 61 - 80 | 25 (27.9) |
| *Median (range), years* | *45 (1-80)* |
|  |  |
| ***Education level:*** |  |
| Never attended school | 31 (32.2) |
| Primary education | 43 (46.2) |
| Secondary education | 19 (21.5) |
| ***Occupation:*** |  |
| Children | 6 (6.5) |
| Students | 9 (9.7) |
| Employed | 9 (9.7) |
| Self employed | 65 (69.9) |
| Unemployed | 4 (4.3) |
| ***Diagnosis:*** |  |
| Infected diabetic wounds | 22 (23.7) |
| Surgical Site Infection | 34 36.6) |
| Infected wounds due to trauma | 25 (26.9) |
| Other wounds | 12 (12.9) |
| ***Type of wound:*** |  |
| Acute wound | 65 (69.9) |
| Chronic wound | 28 (30.1) |
| ***Antibiotic prescriptions (n=82):*** |  |
| Ceftriaxone | 57 (69.5) |
| Metronidazole | 35 (42.7) |
| Cloxacillin | 27 (32.9) |
| Ampiclox | 9 (11.0) |
| Chloramphenical | 3 (3.6) |
| Gentamycin | 3 (3.6) |
| Cristapen | 3 (3.6) |
| Amoxicillin | 1 (1.2) |
