# Supplementary material for: Spectrum and antibiogram of bacteria isolated from patients presenting with infected wounds in a Tertiary Hospital, northern Tanzania
Source: BMC Res Notes. 2017 Dec 20;10:757. doi: 10.1186/s13104-017-3092-9 (PMC5738783; doi:10.1186/s13104-017-3092-9)
Supplement: Supplementary file 2 — Additional file 2: Table S2. Bacteria isolated in different types of wound infection. [file 13104_2017_3092_MOESM2_ESM.docx]

# Table S2: Bacteria isolated in different types of wound infection (N=144).

| **Isolate** | **Type of wound infection**  **n (%)** | | | |  | |
| --- | --- | --- | --- | --- | --- | --- |
|  | **IDFU** | **SSI** | **ITW** | **Other** |  | **Total** |
| S. aureus | 4 (18.2) | 7 (20.6) | 10 (40.0) | 2 (16.7) |  | 23 |
| Coagulase Neg. Staph. | 2 (9.1) | 4 (11.8) | 1 (4.0) | 0 (0.0) |  | 7 |
| Enterococi | 8 (36.4) | 6 (17.6) | 3 (12.0) | 1 (8.3) |  | 18 |
| K. pneumoneae | 5 (22.7) | 3 (8.8) | 1 (4.0) | 2 (16.7) |  | 11 |
| P. aeroginosa | 4 (18.2) | 3 (8.8) | 4 (16) | 4 (33.3) |  | 15 |
| E. coli | 5 (22.7) | 2 (5.9) | 5 (20.0) | 2 (16.7) |  | 14 |
| P. mirabilis | 6 (27.3) | 2 (5.9) | 2 (8.0) | 3 (25.0) |  | 13 |
| Acinetobacter spp. | 1 (4.5) | 2 (5.9) | 1 (4.0) | 0 (0.0) |  | 4 |
| Citrobacter spp. | 1 (4.5) | 0 (0.0) | 1 (4.0) | 0 (0.0) |  | 2 |
| K. oxytoca | 1 (4.5) | 2 (5.9) | 0 (0.0) | 2 (16.7) |  | 5 |
| Other Proteus spp. | 2 (9.1) | 2 (5.9) | 1 (4.0) | 3 (25.0) |  | 8 |
| Other coliforms | 4 (18.2) | 8 (23.5) | 3 (12.0) | 4 (33.3) |  | 19 |
| Beta hemolytic Strept. | 1 (4.5) | 1 (2.9) | 0 (0.0) | 0 (0.0) |  | 2 |
| Viridans Strept | 1 (4.5) | 2 (5.9) | 0 (0.0) | 0 (0.0) |  | 3 |
