# Supplementary material for: Spectrum and antibiogram of bacteria isolated from patients presenting with infected wounds in a Tertiary Hospital, northern Tanzania
Source: BMC Res Notes. 2017 Dec 20;10:757. doi: 10.1186/s13104-017-3092-9 (PMC5738783; doi:10.1186/s13104-017-3092-9)
Supplement: Supplementary file 3 [file 13104_2017_3092_MOESM3_ESM.docx]

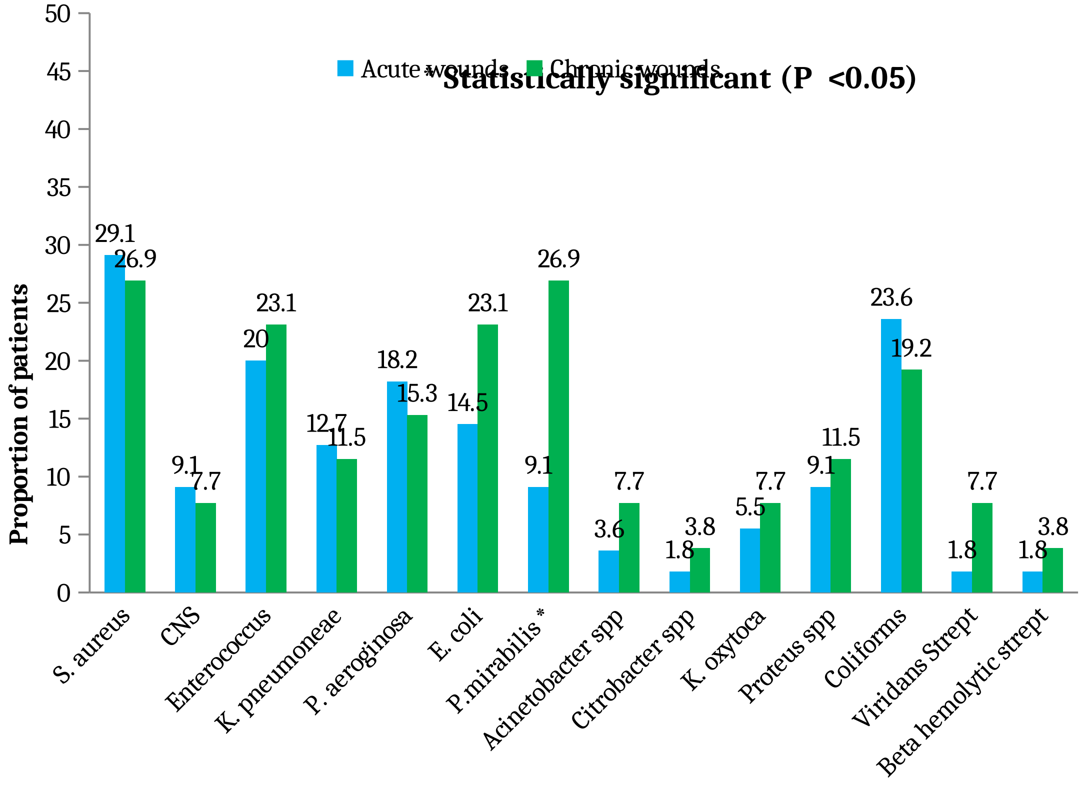

Acute wounds
Chronic wounds
*Statistically significant (P <0.05)
Proportion of patients
50
45
40
35
30
25
20
15
10
5
0
29.1
26.9
9.1
7.7
20
23.1
12.7
11.5
18.2
15.3
14.5
23.1
9.1
26.9
3.6
7.7
1.8
3.8
5.5
7.7
9.1
11.5
23.6
19.2
1.8
7.7
1.8
3.8
S. aureus
CNS
Enterococcus
K. pneumoneae
P. aeroginosa
E. coli
P.mirabilis *
Acinetobacter spp
Citrobacter spp
K. oxytoca
Proteus spp
Coliforms
Viridans Strept
Beta hemolytic strept
